# Exclusion of known corneal dystrophy genes in an autosomal dominant pedigree of a unique anterior membrane corneal dystrophy

**Published:** 2009-08-26

**Authors:** Andrea L. Vincent, David M. Markie, Betina De Karolyi, Catherine E. Wheeldon, Dipika V. Patel, Christina N. Grupcheva, Charles N.J. McGhee

**Affiliations:** 1 Department of Ophthalmology, New Zealand National Eye Centre, Faculty of Medical and Health Sciences, University of Auckland, Auckland, New Zealand; 2 Eye Department, Greenlane Clinical Centre, Auckland District Health Board, Auckland, New Zealand; 3 Department of Pathology, Otago University, Dunedin, New Zealand

## Abstract

**Purpose:**

With advances in phenotyping tools and availability of molecular characterization, an increasing number of phenotypically and genotypically diverse inherited corneal dystrophies are described. We aimed to determine the underlying causative genetic mechanism in a three-generation pedigree affected with a unique anterior membrane corneal dystrophy characterized by early onset recurrent corneal erosions, small discrete focal opacities at the level of Bowman layer and anterior stroma, anterior stromal flecks, and prominent corneal nerves.

**Methods:**

Twenty affected and unaffected members of a three-generation family were examined and extensively clinically characterized including corneal topography and in vivo confocal microscopy, and biological specimens were collected for DNA extraction. Mutational analysis of two corneal genes (*TGFBI* [Transforming Growth factor-beta induced] and *ZEB1* [zinc finger E box-binding homeobox 1]) was undertaken, in addition to testing with the Asper Corneal Dystrophy gene chip (Asper Ophthalmics, Tartu, Estonia). Subsequent Genotyping To 11 Known Corneal Gene Loci (*COL8A2* [Collagen, Type VIII, Alpha-2], *TACSTD2* [Tumor-Associated Calcium Signal Transducer 2], *PIP5K3* [Phosphatidylinositol-3-Phosphate 5-Kinase, Type III], *GSN* [Gelsolin], *KERA* [Keratocan], *VSX1* [Visual System Homeobox Gene 1], *COL6A1* [Collagen, Type VI, Alpha-1], *MMP9* [Matrix Metalloproteinase 9], *KRT3* [Keratin 3]), and two putative loci, 3p14-q13 and 15q22.33–24) was undertaken using polymorphic markers, and haplotypes constructed. Multipoint linkage analysis was performed to generate LOD scores and produce LOD plots across the candidate intervals.

**Results:**

No pathogenic sequence variations were detected in *TGFBI* or *ZEB1* of the proband nor on the Asper Corneal Dystrophy gene chip (302 mutations in 12 genes). Multipoint linkage analysis of 11 known corneal genes and loci generated negative LOD plots and was able to exclude all genes tested including *PIP5K3.*

**Conclusions:**

Exclusion of linkage to candidate corneal loci combined with an absence of pathogenic mutations in known corneal genes in this pedigree suggest a different genetic causative mechanism in this dystrophy than the previously documented corneal genes. This unique phenotype of an anterior membrane dystrophy may therefore provide an opportunity to identify a new gene responsible for corneal disease.

## Introduction

The inherited corneal dystrophies represent a group of clinically and genetically heterogeneous disorders of the cornea, with many genes identified to date. With the advances in phenotyping tools and the availability of molecular characterization, an increasing amount of phenotypic and genotypic diversity is described in the corneal dystrophies. The autosomal dominantly inherited bilateral corneal dystrophies involving the anterior corneal layers of the epithelial basement membrane, Bowman layer, and/or anterior stroma are typically described as occurring in association with *TGFBI* (Transforming Growth factor-beta induced) [1] or *PIP5K3* (Phosphatidylinositol-3-Phosphate 5-Kinase, Type III) [2] mutations. The *TGFBI* dystrophies (granular, lattice, granular dystrophy type 2, and Bowman layer [Reis-Bückler, Thiel-Behnke]) are well characterized and frequently demonstrate typical and reproducible phenotype-genotype correlations, yet there are an increasing number of new phenotypic and genotypic descriptions that are not compatible with the current forms of corneal dystrophies [1].

Less is published on Fleck corneal dystrophy (CFD; OMIM 121850), a term characteristically used in reference to the dystrophy initially described by François and Neetens as “heredodystrophie Mouchetee [2],” which is translated as “speckled.” Multiple flecks are predominantly located centrally and peripherally in the stroma with clear normal tissue intervening and no involvement of other layers of the cornea, including Bowman layer. The disease is usually non-progressive, and the flecks are typically seen by slit-lamp biomicroscopy to be predominantly in the posterior corneal stroma. The majority of patients are asymptomatic. Recurrent erosions are not a typical feature, although photophobia is reported [3,4]. In vivo confocal microscopy of fleck dystrophies highlights small bright deposits in and around keratocyte nuclei that occur throughout the stroma despite the clinical appearance [5,6].

Four families with classic Fleck dystrophy demonstrated linkage to chromosome 2q35 between D2S117 and D2S116 with a maximum multipoint LOD score of 5 halfway between D2S2289 and D2S325 [7]. Subsequent sequencing of candidate genes within the interval between D2S2289 and D2S126 identified pathogenic mutations in *PIP5K3* in 8 of 10 families with Fleck dystrophy [8], although incomplete penetrance was documented in some families. *PIP5K3* encodes for a member of the phosphoinositide 3-kinase family and is involved in the regulation of endosomal transport. However, it must be noted that in addition to being dystrophic, corneal flecks may also be degenerative, a feature of certain systemic or topical drugs, or associated with contact lens wear [5,9].

We have identified a three-generation family with a unique corneal dystrophy demonstrating autosomal dominant inheritance and presenting with frequent, recurrent corneal erosions in the first decade but with a relatively mild clinical appearance. This phenotype is not that of the classic (posterior) stromal Fleck dystrophies, and while it clearly exhibits multiple anterior stromal flecks, the corneal localization of larger circular/oval opacities at the level of Bowman layer and anterior stroma suggests an anterior membrane type dystrophy. This pedigree is assumed dystrophic predominantly because of the inheritance, bilaterality, and exclusion of long-term and/or recent contact lens wear.

We aimed to determine the causative genetic mechanism for this disease by undertaking mutational analysis of candidate corneal genes and linkage analysis to known corneal genes and loci.

## Methods

**Figure 1:**
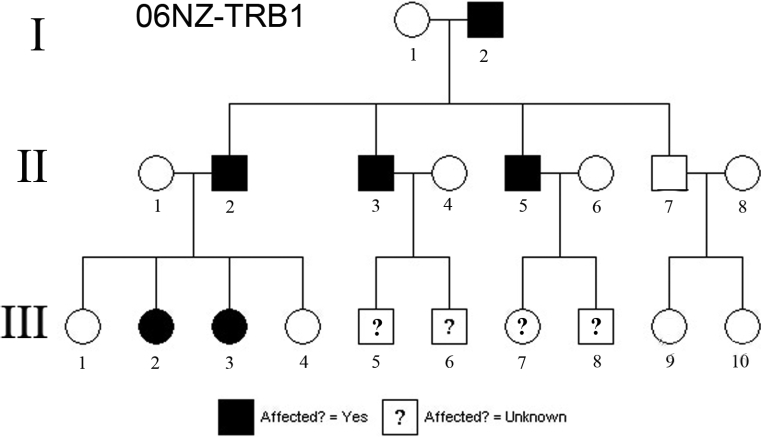
Pedigree of the reported family. Individuals III:5, III:6, III:7, and III:8 are younger than the average age of onset of symptoms, therefore affectation status can not be assigned.

Following informed consent, 20 members of the three-generation family (Figure 1) were recruited, and all members were characterized clinically with visual acuity, autorefraction, slit-lamp biomicroscopic examination, and Orbscan II slit-scanning elevation topography (Bausch & Lomb Surgical, Rochester, NY). Selected individuals also underwent in vivo confocal microscopy (Confoscan 2; Fortune Technologies, Greensboro, NC and/or Heidelberg Retina Tomograph II, Rostock Corneal Module [RCM]; Heidelberg Engineering GmbH, Heidelberg, Germany). This study adhered to the principles of the Declaration of Helsinki and received institutional ethics approval (AKX0200002 and NTX0612161).

Since the clinical manifestation of the disease is relatively mild, being restricted to corneal erosive episodes without visual impairment, none of the affected individuals have undergone corneal biopsy or penetrating keratoplasty. Therefore, histological analysis was unavailable.

Biological samples (peripheral venous blood or saliva specimen) were collected for DNA extraction, which was obtained using the salt extraction method from blood [10] and according to the manufacturer’s instructions for saliva specimens (Oragene^TM^; DNAGenotek, Ottawa, ON, Canada). Mutational analysis of all 17 coding exons of *TGFBI* was undertaken using previously described primers and methods [11,12].

Recurrent corneal erosions in a patient with epithelial basement membrane dystrophy have been described as occurring concurrently with posterior polymorphous dystrophy (PPD) [13]. Localized bullous epithelial changes are also described occurring in association with PPD [14]. Mutational analysis of nine coding exons of *ZEB1* (zinc finger E box-binding homeobox 1), formerly *TCF8*, (transcription factor 8) a corneal gene implicated in PPD, was also undertaken using previously described primers and methods. For exon 1; primers were from Aldave et al. [15], for exons 2-9 primers were from Krafchak et al. [16]. Polymerase chain reaction (PCR) amplification was undertaken using Roche Faststart polymerase (F. Hoffman-La Roche Ltd., Basel, Switzerland). The amplification cycles were 95 °C for 5 min, then 30 cycles of 95 °C for 30 s, annealing temperature (varied by primer pair) for 1 min, 72 °C for 1min. A final extension step of 72 °C for 10 min then completed the PCR reaction. Product was visualised on a 1.5% agarose gel. For sequencing, the products were purified with Roche HighPure PCR product purification kit (F. Hoffman-La Roche Ltd.), and then sequenced using the same primer pair. Sequences were examined manually and with CLC DNA workbench (CLCbio, Aarhus, Denmark) and aligned with genomic DNA reference: NC_000010.10 and mRNA reference sequence NM_030751.4.

A DNA sample of the proband was sent to Asper Ophthalmics (Tartu, Estonia) and tested against the Corneal Dystrophy chip, Version 2.0 (Asper Ophthalmics). A review of the literature identified known corneal genes and loci implicated in the pathogenesis of corneal disease, and bioinformatic databases were used to identify flanking and intragenic polymorphic microsatellite markers for each known gene. For these candidate loci, polymorphic markers were selected to cover the interval. If one marker failed to adequately amplify or demonstrated low informativeness in the family, a further marker was identified. The loci and markers used are shown in Table 1.

**Table 1:** Candidate corneal genes and loci.

**Gene or locus**	**Chromosomal location**	**Number of markers**
*COLVIIIA2*	1p34.3-p32	3
*TACSTD2*	1p32–31	3
*PIP5K3*	2q33.3	10
*KTCN3* locus	3p14-q13	6
*GSN*	9q34	3
*KERA*	12q22	3
*KRT3*	12q12-q13	3
Hughes Keratoconus [25,26]	15q22.33–24.2	8
*VSX1*	20p11-q11	3
*MMP9*	20q12	4
*COLVIA1*	21q22.3	3

For each candidate gene or locus of interest (column 1) the chromosomal location is given (column 2). Also for each locus the number of polymorphic microsatellite markers used for linkage analysis across each interval is stated in column 3. Abbreviations in the table are: *COLVIIIA2* = Collagen, Type VIII, Alpha-2, *TACSTD2* = Tumor-Associated Calcium Signal Transducer 2, *PIP5K3* = Phosphatidylinositol-3-Phosphate 5-Kinase, Type III, KTCN3 = keratoconus 3, *GSN* = Gelsolin, *KERA* = Keratocan, *KRT3* = Keratin 3, *VSX1* = Visual System Homeobox Gene 1, *MMP9* = Matrix Metalloproteinase 9, *COLVIA1* = Collagen, Type VI, Alpha-1.

Primer pairs were synthesized and PCR amplification undertaken using Roche Faststart polymerase (F. Hoffman-La Roche Ltd.). The amplification cycles were as described for *TCF8*/*ZEB1* above, with annealing temperature varying for each primer pair in order to optimise the amount of product. Samples were then run on ABI3700 Genotyper, (Applied Biosystems Inc., Foster City, CA), and ABI software was used to assign the peaks. Haplotypes were constructed, and multipoint linkage analysis using GeneHunter v2.1 [17] was undertaken to generate LOD plots across the candidate regions.

## Results

The clinical features of members of this pedigree are summarized in Table 2.

**Table 2:** Individual patient demographics and examination findings.

Patient ID	Sex	Affected status	Age at last exam	Age at onset	Age at offset	Visual acuity		Pachymetry		Anesthesiometry
						RE	LE	RE	LE	
I-1	F	Unaffected	65	N/A	N/A	6/6	6/15-1	568	569	ND
I-2	M	Affected	64	‘childhood’	unsure	6/7.5-1	6/7.5-	poor	reading	0.76
II-2	M	Affected	41	10 years	21	6/12	6/7.5	559	558	0.82
II-3	M	Affected	39	6 years	13	6/6+	6/6	550	552	0.38
II-5	M	Affected	38	5 years	22	6/6	6/6+1	513	491	0.78
II-7	M	Unaffected	37	N/A	N/A	6/6	6/5	554	549	0.42
III-1	F	Unaffected	15	N/A	N/A	6/9	6/6	566	558	0.29
III-2	F	Affected	14	5 years	ongoing	6/5	6/5	542	540	0.41
III-3	F	Affected	12	5 years	ongoing	6/5	6/5-1	558	549	0.26
III-4	F	Unaffected	11	N/A	N/A	6/5-	6/7.5-	ND	ND	0.28
III-5	M	Unknown	5	Nil to date	N/A	6/6	6/7.5-	ND	ND	ND
III-6	M	Unknown	4	Nil to date	N/A	6/6	6/6	ND	ND	ND
III-7	F	Unknown	10	Nil to date	N/A	6/5-2	6/6+	ND	ND	ND
III-8	M	Unknown	5	Nil to date	N/A	6/6-2	6/6-	ND	ND	ND

Table 2 lists the demographic features for each individual in the pedigree including sex (F = female, M = male), age at last examination in years, affectation status, and age in years at onset and offset of the recurrent corneal erosions for those affected. For those unaffected this information is not applicable (N/A). Clinical examination findings listed include visual acuity measured with the Snellen chart, pachymetry indicating central corneal thickness measured in microns, and anaesthesiometry, units are in millibars. When the information was not available, it is listed in the table as Not Done (ND).

Affected individuals typically presented in childhood at an average age of six years with recurrent corneal erosions (RCEs), occurring approximately every two to three months. These RCEs became much less frequent in the third and fourth decade, indeed, with increasing age, the recurrent epithelial erosions became less frequent, and the episodes appeared to largely “burn out” by early to mid 20s. A few of the older adults reported photophobia and foreign body sensation and continued to use topical ocular lubricants frequently for ocular comfort. Due to their very young age, four individuals in the youngest generation could not yet be assigned affectation status.

**Figure 2:**
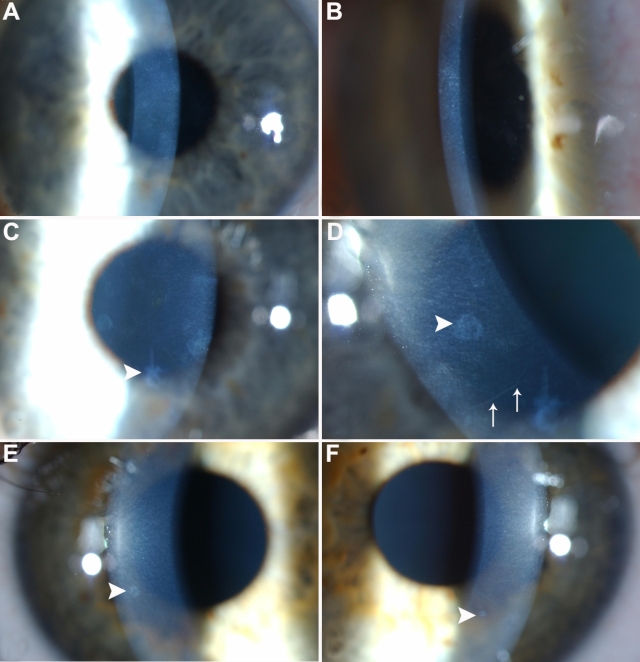
Slit lamp images of corneas of affected family members. Slit lamp images of cornea highlighting diffuse, small, gray anterior stromal flecks (**A-F**) and larger, discrete circular/oval/annular gray/white opacities (0.2–1.5 mm) at the level of Bowman's layer (**C-F**, small arrowheads) and prominent corneal nerves (**D**, fine arrow). **A**: Slit lamp image of right cornea of individual I:2, age 64 years, at 10× magnification, demonstrating diffuse small gray anterior stromal flecks and annular opacities. **B**: Slit lamp image of left cornea of individual II:5, age 37 years, at 10× magnification demonstrating diffuse small gray anterior stromal flecks and annular opacities. **C **and **D**: Slit lamp images of right cornea of individual II:3, at 10× magnification (**C**) and 16×  magnification (**D**). The annular opacities are clearly visualised (small arrowhead) with prominent corneal nerves visible (fine arrow; **E** and **F**) Slit lamp images of right and left corneas respectively of individual III:2, age 14 years, at 10× magnification showing the annular opacities highlighted with arrowheads.

Three slit-lamp biomicroscopic corneal features were common to the six affected individuals: (1) accumulation of small, variable size (0.2–1.5 mm diameter), discrete, grayish-white oval-round or annular opacities at the level of Bowman layer and the superficial anterior stroma (typically less than six lesions per cornea; Figure 2); (2) numerous, prominent small gray flecks in the anterior 20%–25% of the stroma extending from the central cornea to the limbus; and (3) five of the six affected subjects also had prominent corneal nerves (with reduced corneal sensation more prevalent in the older members of the pedigree). The lesions identified appeared on a background of a generally clear translucent cornea with no involvement of the deeper stroma, Descemet membrane, or the endothelium, and there was an absence of neovascularization.

**Figure 3:**
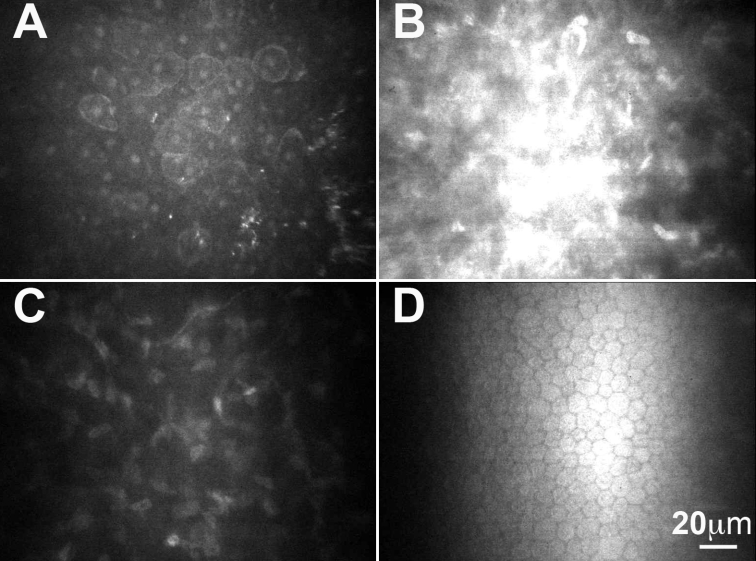
In vivo confocal microscopy (IVCM): Confoscan 2 images of I:2. **A**: IVCM image of normal epithelium with prominent nuclei visible. **B**: IVCM image at the level of Bowman membrane demonstrating abnormal diffuse hyper-reflectivity. **C**: IVCM image at the level of the posterior stroma demonstrating a normal keratocyte appearance with no evidence of hyper-reflective structures (flecks) either within the keratocytes, or in the extracellular regions. **D**: IVCM image at the level of the endothelium, demonstrating normal healthy endothelium.

**Figure 4:**
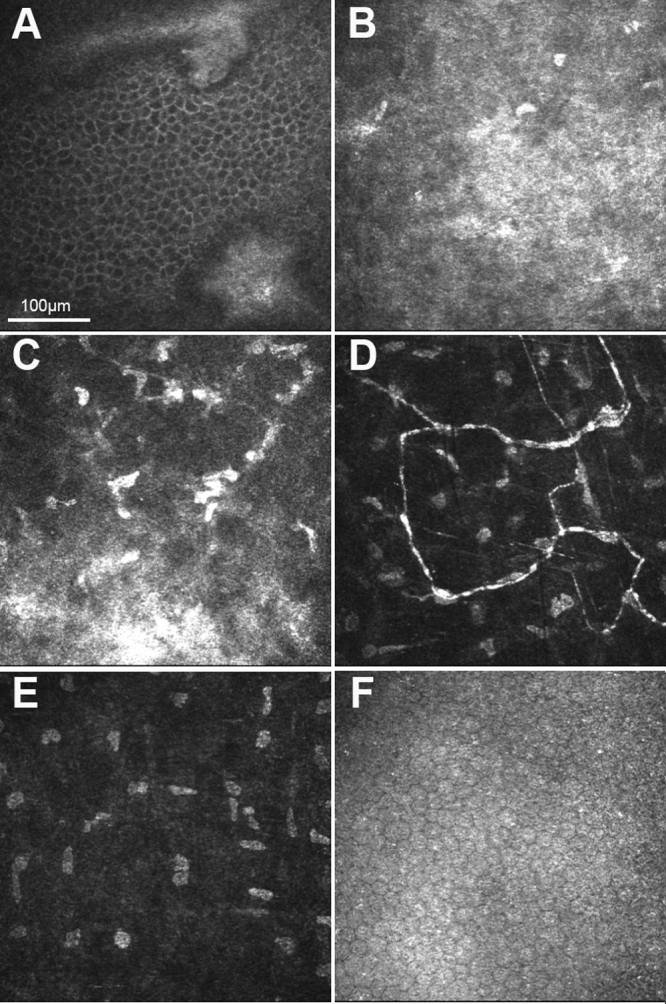
In vivo confocal microscopy (IVCM): Heidelberg Retina Tomograph II, Rostock Corneal Module images of individual II:3. **A**: IVCM image at the level of the basal epithelium, demonstrating two irregular amorphous regions of Bowman layer protruding forwards into the epithelium, visible in the upper and lower aspect of the image. **B**: IVCM image at the level of Bowman layer, demonstrating a diffuse, irregular hyper-reflectivity. Sub-basal nerves are not visible. **C**: IVCM image at the level of the anterior stroma, demonstrating patchy areas of hyper-reflectivity within the stroma with hyper-reflectivity of keratocytes. **D**: IVCM image at the level of the midstroma, demonstrating unusual, tortuous stromal nerves with normal keratocytes. **E**: IVCM image at the level of the posterior stroma demonstrating normal architecture and normal keratocytes. **F**: IVCM image at the level of the endothelium, demonstrating normal healthy endothelium.

Orbscan II slit-scanning elevation topography (Bausch & Lomb Surgical) of affected individuals demonstrated normal keratometric patterns and pachymetry. Subsequent in vivo confocal microscopy confirmed that the corneal opacities were restricted to the area of Bowman layer and anterior stroma (Figure 3 and Figure 4), and there appeared to be no abnormalities of the anterior keratocytes or stromal deposits consistent with the clinical appearance of multiple diffuse anterior stromal flecks.

Three clinically unaffected individuals demonstrated faint stromal flecks of size consistent with keratocytes (I:1, II:7, III:7). None of the clinically unaffected individuals demonstrated the Bowman layer circular/oval opacities observed in the affected subjects.

No pathogenic mutations were identified in any of the 17 coding exons and flanking intronic regions of *TGFBI *or in the nine coding exons and flanking intronic regions of *ZEB1*. Testing of an affected individual’s DNA against the Asper Corneal Dystrophy microarray Version 2.0 (302 mutations in 12 genes: *COL8A2* (Collagen, Type VIII, Alpha-2), *TGFBI*, *VSX1* (Visual System Homeobox Gene 1), *CHST6* (Carbohydrate Sulfotransferase 6), *KRT3* (Keratin 3), *KRT12* (Keratin 12), *GSN* (Gelsolin),* TACSTD2* (Tumor-Associated Calcium Signal Transducer 2), *CYP4V2*, (Cytochrome P450, Family 4, Subfamily V, Polypeptide 2)* SOD1* (*S*uperoxide Dismutase 1;), *ZEB1* and *SLC4A11* (Solute Carrier Family 4 [Sodium Borate Cotransporter], Member 11) revealed a non-pathogenic heterozygous sequence change in *CHST6* (c.484C>G), resulting in p.R126G but no pathogenic mutations.

**Figure 5:**
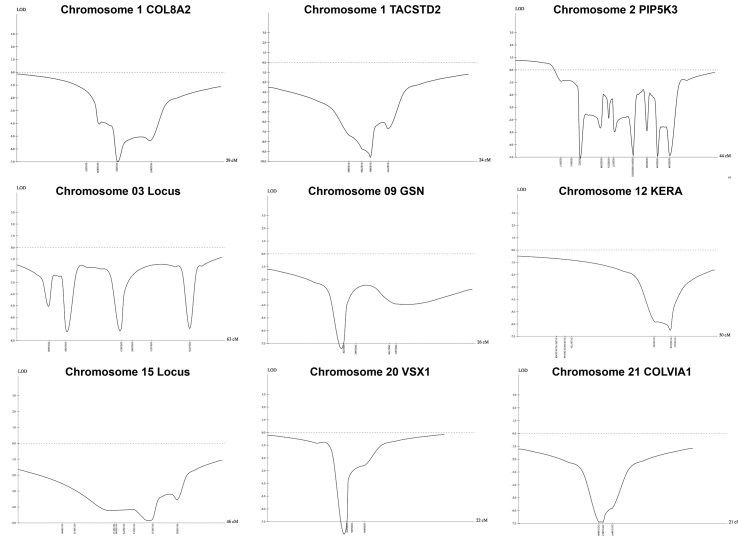
LOD plots. Multipoint linkage LOD plots of candidate genes and regions are demonstrated. All plots within the candidate interval fall below zero and have negative LOD scores.

Multipoint linkage analysis of nine genes and loci shown in Table 1 was predominantly negative across the intervals. Although the maximum LOD score achievable in this family with the current information is 2.1, the LOD plots generated by multipoint linkage analysis (Figure 5) suggest that all tested candidate loci are excluded or highly unlikely to be responsible.

## Discussion

This pedigree consisted of three generations with six affected individuals that clearly demonstrate autosomal dominant inheritance. The clinical phenotype in this pedigree is of early onset recurrent erosions associated with an unusual anterior membrane/fleck dystrophy. Some features are similar to the original description in 1966 of an eponymous entity, Grayson Wilbrandt dystrophy (GWD) [18]. GWD varies from the classic fleck description– GWD was described with infrequent erosions, and a less severe effect on vision than occurred in the Bowman layer dystrophies. The original clinical description of GWD is of gray-white amorphous opacities of various sizes over the central cornea, consisting of mounds extending into the epithelium from a thickened Bowman layer with a clear intervening stroma. Corneal nerves were described as prominent.

Further characterization of corneal dystrophies, particularly fleck dystrophy, can be achieved using in vivo confocal microscopy (IVCM) with typical findings consisting of hyper-reflective spots within pleomorphic keratocyte nuclei as previously described [5,6,19].

The phenotypic presentation in the pedigree here particularly with frequent erosions is more in keeping with a *TGFBI* dystrophy. Given the spectrum of phenotypic variability demonstrated in the *TGFBI*-associated dystrophies to date with the presence of photophobia and recurrent corneal erosions in many of the family members, mutational analysis of *TGFBI* was believed to be a valid first approach. Interestingly, *TGFBI* analysis failed to demonstrate any pathogenic mutation.

Mutational analysis using the corneal dystrophy disease microarray (Asper Ophthalmics) tested for all known mutations in addition to single nucleotide polymorphisms (SNPs) in 12 corneal genes, but none of the known mutations were identified in the proband. As no *TGFBI* mutation was identified in this pedigree and the phenotype is a “unique” dystrophy, genotyping, haplotype construction, and multipoint linkage to candidate loci and genes including the Fleck locus on 2q35 (where subsequently *PIP5K3* was identified as the causative gene) were undertaken. This method is useful to determine if any candidate genes warrant sequencing including *PIP5K3*, a very large gene of 41 exons encompassing more than 89 kb.

*PIP5K3* is a member of the phosphoinositide 3-kinase family and regulates the sorting and traffic of peripheral endosomes that contain lysosomally directed fluid phase cargo by controlling the morphogenesis and function of multivesicular bodies.

Limitations of the methods used for gene or loci exclusion include the Asper Corneal Dystrophy microarray only excluding known mutations in known genes. As the genes not excluded by microsatellite analysis and linkage (e.g., *CHST6*, *KRT12*, *SLC4A11*, and *SOD1*) were not fully sequenced, it is feasible that a unique mutation may reside on one of the candidate genes. Similarly, sequencing of *TGFBI* did include all coding exons and flanking intronic regions but, theoretically, would not detect copy number variants within the *TGFBI* open reading frame (ORF).

This dystrophy shows some similarities to two recently described autosomal dominant dystrophies with recurrent corneal erosions, Dystrophia Helsinglandica [20] and the Dystrophia Smolandiensis variant [21]. However, Dystrophia Helsinglandica gradually manifests with subepithelial fibrosis, and the Dystrophia Smolandiensis variant results in keloid-like corneal nodules with penetrating keratoplasty often being required. There are also some similarities to subepithelial mucinous corneal dystrophy (SMCD), although more significant vision loss resulted in the one SMCD family described [22]. Franceschetti [23] described a pedigree with RCE in 1928. However, as this original description is limited to one boy, it is difficult to determine similarities. Using the IC3D (International Committee for Classification of Corneal Dystrophies) classification [1], this anterior membrane dystrophy falls in the descriptive category 4: “This category is reserved for a suspected new, or previously documented corneal dystrophy, although the evidence for it, being a distinct entity, is not yet convincing” [1].

By resolving the affectation status of the four children in the youngest generation (III-5,III-6, III-7, and III-8), the statistical power increases so as to achieve a potential LOD score of around 3.3, suitable for providing positive gene localization in a genome-wide linkage study. This phenotype has an age-dependent penetrance, so until all of these younger children reach 10 years of age, one cannot confidently label them as unaffected. Non-penetrance may also be an issue as has been described occurring in fleck dystrophy associated with *PIP5K3 *mutations [8] and in some of the *TGFBI* – associated corneal dystrophies [24].  This issue may reduce somewhat the power of linkage based approaches in this family.

Thus, to genetically characterize this family further, ongoing clinical examination and observation of the younger and clinically unaffected members of the pedigree must be undertaken. Once affectation status can be more tightly assigned, it may be possible to obtain a higher potential LOD score, which will permit a tighter search of the genome. This could be rapidly achievable using SNP microarrays such as the Affymetric Gene chip systems (Affymetrix, Santa Clara, CA). The clinical findings, analysis of linkage, and mutational analysis suggest this family may provide a clue to a novel genetic mechanism and/or genes implicated in the pathogenesis of the dominantly inherited anterior corneal dystrophies.
